# Retraction Note: Reduction of carbon dioxide to oxalate by a binuclear copper complex

**DOI:** 10.1038/s41467-021-21951-5

**Published:** 2021-03-25

**Authors:** Uttam R. Pokharel, Frank R. Fronczek, Andrew W. Maverick

**Affiliations:** Department of Chemistry, Louisiana State University, Baton Rouge, Louisiana 70803 USA

Retraction of: *Nature Communications* 10.1038/ncomms6883, published online 19 December 2014.

During follow-up work to this Article, the authors discovered that its main claim—namely, that CO_2_ was reduced to form oxalate—is incorrect. Rather, the detected oxalate is formed by the oxidation of ascorbate in the system, as discussed in a subsequent analysis^1^. Although other results reported therein are valid, the authors wish to retract this Article.
